# Second line trastuzumab emtansine following horizontal dual blockade in a real-life setting

**DOI:** 10.18632/oncotarget.27603

**Published:** 2020-06-02

**Authors:** Salvatore Del Prete, Liliana Montella, Grazia Arpino, Giuseppe Buono, Carlo Buonerba, Pasquale Dolce, Olga Fiorentino, Maria Aliberti, Antonio Febbraro, Clementina Savastano, Giuseppe Colantuoni, Ferdinando Riccardi, Angela Ruggiero, Sabino De Placido, Michele Orditura

**Affiliations:** ^1^ Medical Oncology Unit “San Giovanni di Dio” Hospital, Frattamaggiore, Naples 80027, Italy; ^2^ Medical Oncology Unit “Santa Maria delle Grazie” Hospital, Pozzuoli, Naples 80078, Italy; ^3^ Department of Clinical Medicine and Surgery, Oncology Division, University of Naples Federico II, Naples 80131, Italy; ^4^ Department of Public Health, University of Naples Federico II, Naples 80131, Italy; ^5^ Medicina Futura Group, via Alcide de Gasperi, Acerra, Naples 80011, Italy; ^6^ Medical Oncology Unit, Hospital Sacro Cuore di Gesù Fatebenefratelli, Benevento 82100, Italy; ^7^ Medical Oncology Unit, San Giovanni di Dio e Ruggi d’Aragona, Salerno 84121, Italy; ^8^ Medical Oncology Unit, San Giuseppe Moscati Hospital, Avellino 83100, Italy; ^9^ Medical Oncology Unit, Antonio Cardarelli Hospital, Napoli 80131, Italy; ^10^ Division of Medical Oncology, Department of Precision Medicine, School of Medicine, University of Study of Campania “Luigi Vanvitelli”, Naples 80131, Italy

**Keywords:** breast cancer, metastatic, Her-2 positive, trastuzumab emtansine, pertuzumab

## Abstract

Despite relevant medical advancements, metastatic breast cancer remains an uncurable disease. HER2 signaling conditions tumor behavior and treatment strategies of HER2 expressing breast cancer. Cancer treatment guidelines uniformly identify dual blockade with pertuzumab and trastuzumab plus a taxane as best first line and trastuzumab emtansine as preferred second line choice. However, there is no prospectively designed available study focusing on the sequence and outcomes of patients treated with T-DM1 following the triplet. In the following report, data concerning a wide series of patients treated in a real-life setting are presented. Results obtained in terms of response and median progression free survival suggests a significant role for T-DM1 in disease control of metastatic HER2 expressing breast cancer.

## INTRODUCTION

From 80s, Human Epidermal Growth Factor Receptor 2 (HER2) signaling was increasingly recognized as pivotal in tumor growth of HER2 expressing breast cancer. HER2 expression is limited to a proportion (15–20%) of breast cancer; however, HER2 conditions tumor behavior and addresses treatment strategies. Current available guidelines in metastatic HER2 positive breast cancer designs a sequence of treatment with first-line double blockade with trastuzumab plus pertuzumab and a taxane according to Cleopatra trial results [1] and second-line treatment with trastuzumab emtansine (T-DM1) enforced by Emilia trial results [2] and, then, lapatinib plus capecitabine [3].

T-DM1 is an antibody-drug conjugate, which drives the chemotherapeutic maytansine directly to HER2 expressing cells through the driver trastuzumab [4]. The development of drugs like T-DM1 arise from the concept of increasing drug concentration at the tumor and mitigated toxicity due to drug deliver at the site of HER2 expressing tumor cells. Therapy with antibody-drug conjugates is a tempting strategy especially in later lines of treatment as showed by recent results with trastuzumab deruxtecan (DS-8201) [5]. Since T-DM1 introduction in clinical practice in second-line, overall survival has reached 30.9 months [2]. The reduced toxicity of T-DM1 in second and later lines of treatment together and the high rates of activity and efficacy [2] are determinant in choosing treatment for a patient candidate to a prolonged time on treatment. On February 22, 2013, the Food and Drug Administration (FDA) approved T-DM1 for use as a single agent in the treatment of patients with HER2-positive metastatic breast cancer who previously received trastuzumab and a taxane [6]. In 2019, the KATHERINE trial showed a significant benefit of adjuvant T-DM1 in HER2-positive patients treated with neoadjuvant treatment and not achieving a pathologic complete response [7]. In fact, these patients showed with adjuvant T-DM1 a risk of recurrence 50% lower than with trastuzumab alone. Such results induced FDA on May 3, 2019 to approve T-DM1 for the adjuvant treatment of patients with HER2-positive early breast cancer treated with neoadjuvant taxane and trastuzumab-based treatment and having residual invasive disease in the breast or axilla at surgery [8]. In Italy, T-DM1 is authorized in this setting on an individual basis in the context of a compassionate use.

From publication of Emilia results, T-DM1 has been considered the preferred choice for patients progressing on trastuzumab and results superior to lapatinib and capecitabine [2, 9].

In preclinical models, T-DM1 potently inhibits growth of trastuzumab-sensitive and -insensitive HER2-amplified cancer cells [10]. Despite impressive results shown by T-DM1, mechanisms of resistance could occur. Among these mechanisms there are hindrance of trastuzumab binding to HER2 caused by mucin 4 (MUC4) expression, defects in intracellular metabolism of T-DM1 owing to impaired lysosomal proteolytic activity, efflux of DM1 as a result of the expression of multidrug resistance (MDR) transporters [11]. Moreover, truncated HER2 (p95HER2), which lacks binding sites to trastuzumab and pertuzumab and maintains the kinase domain, results in resistance to trastuzumab, pertuzumab, and T-DM1 [12].

In the present study, data coming from different centers concerning patients with HER2 positive metastatic breast cancer treated with second line T-DM1 following trastuzumab and pertuzumab were collected and evaluated. T-DM1 is suggested as preferred choice in second line treatment by different guidelines [13–15], however, there are contradictory reports suggesting either less activity in second-line after pertuzumab plus trastuzumab combination [16] or decreased activity in later line of treatment [17]. While prospective studies addressing the issue of second line with T-DM1 probably will not arrive because the sequence of first and second line in this setting is standard practice, different experiences have been reported [18–21]. They can be concordant or discordant as concerns some discussion points, but they comprehensively contribute to suggest a likely response. In line with the previous studies, the reported multicenter experience has retrospectively evaluated activity, duration of response, progression free survival, safety and clinical and pathological factors potentially influencing these outcomes in a series of patients with metastatic HER2 positive breast cancer treated with second-line T-DM1 following pertuzumab, trastuzumab and docetaxel in first-line.

## RESULTS

### Study population

One hundred thirty-five patients (age range 34-87, median age 56 years) corresponding to the predefined criteria were screened in the seven centers invited to participate. Baseline patients’ characteristics are represented in Table 1.

**Table 1:** Baseline patients’ characteristics

Number of patients	135
Age (range, median)	34-87, 56 years
Hormone-receptor	
-positive	91/135 (67%)
-negative	44/135 (33%)
HER2 3+ (IHC)	106
HER2 positive (FISH)	29
Ki 67 ≥ 20%	105/135 (78%)
< 20%	26/135 (19%)
unknown	4/135 (3%)
Pre-menopausal	64 (47%)
Metastasis at diagnosis	47 (35%)
Metastasis at time of first-line treatment	
- Visceral	84/135 (64%)
Brain metastases	15/135 (11%)
Adjuvant trastuzumab	65/135 (48%)
First line treatment with pertuzumab plus trastuzumab	100%

Patients were almost equally distributed by menopausal status, being 53% post- and 47% pre-menopausal. Hormone-receptor positive tumors were predominant and represented 67% of the patients. In most of the cases, HER2 assessed as 3+ came from (about 78%). In cases resulting HER 2+ at IHC, Fluorescent in Situ Hybridization (FISH) was needed to define HER2 as positive. Tumors were classified as Ki67 ≥ 20% in most of the cases (78%). About half of the patients (48%) have been pretreated with adjuvant trastuzumab. In 35% of the patients, metastases were present at initial evaluation. Visceral metastatic involvement was recognized in 64% of the patients at time of first-line treatment. Brain metastases were discovered in 11% before second-line therapy.

### Outcome measures

Table 2 summarizes study results. Overall Response Rate (ORR: Complete plus Partial Responses) to first and second-line treatment were respectively 42% and 20.7%. Median duration of first-line treatment was 469 days (about 15.6 months). Focusing on results to second-line treatment, 5 (about 4%) and 23 patients (17%) reported respectively complete and partial response to second-line treatment. These patients also reported a long-lasting response in a wide majority of cases (about 68% of cases). Interestingly, clinical benefit rate reaching 50% was similar in first and second line. Sixty out of 135 patients (44%) remained on treatment with T-DM1 more than one year. Among 10 patients progressing on first line triplet, prolonged disease stabilization was reported in three patients. At the data cut-off (October 30, 2019; median follow-up: 309 days, about 10,3 months) 28 out of 135 patients (20%) stayed on treatment while the remaining stopped trastuzumab emtansine due to disease progression.

**Table 2:** Results

Best response to first-line treatment	
- CR	4/135 (3%)
- PR	53/135 (39%)
- SD	67/135 (50%)
- PD	10/135 (7,4%)
- UK	1/135 (0,7%)
- ORR	42%
- Clinical benefit rate (CR+PR+SD ≥6 months)	68/135 (50%)
Best response to second-line treatment	
- CR	5/135 (3,7%)
- PR	23/135 (17%)
- SD	73/135 (54%)
- SD ≥6 months	50/135 (37%)
- PD	24/135 (17,7%)
- UK	10/135 (7,4%)
- ORR	20,7%
- Clinical benefit rate (CR+PR+SD ≥6 months)	57,7%
PFS (range, months)	10,5

Legend: CR complete response, PR partial response, SD stable disease, UK unknown, ORR overall response rate, PFS: Progression Free Survival.

**Figure 1:**
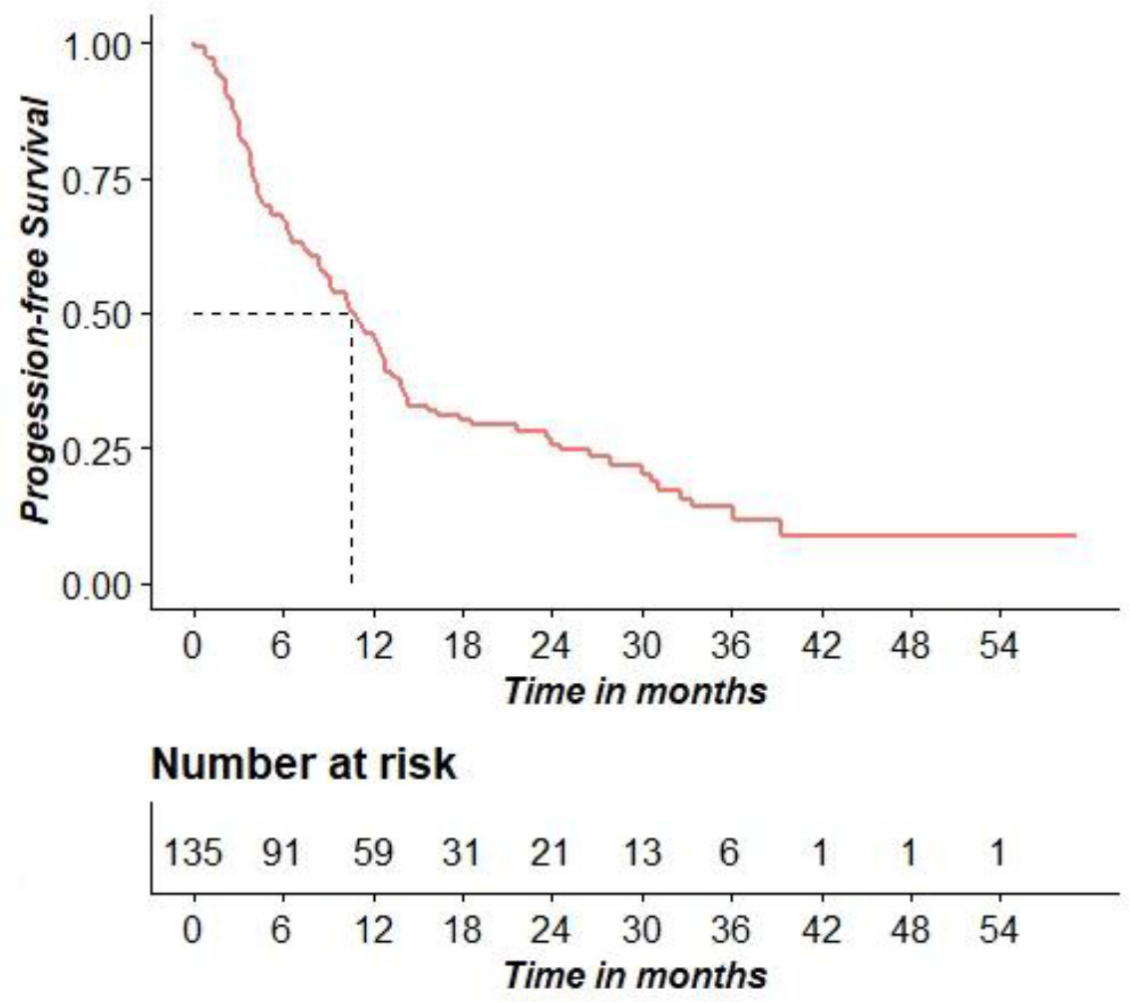
Kaplan–Meier curve for PFS.

At the data of analysis, the proportion of patients dead and alive were almost equally distributed. Median progression free survival (mPFS) was 315 days (10.5 months), (95% C. I. [258; 382] days, [8.6; 12.7] months). Figure 1 shows the PFS probability distribution.

### Evaluation of factors with potential influence on outcome measures

Univariate Cox regression analysis showed that only age more than 56 years old and menopausal status were associated to an increased risk of progression (Table 3). However, adjusting for the effects of the other variables, only menopause showed a significant association with PFS (HR = 1.97, 95%CI [1.16; 3.34], *p* = 0.012).

**Table 3:** Cox regression model analysis

	Progression		Univariate Analysis		Multivariate Analysis	
	Yes (*n* = 106)	No (*n* = 29)	HR	*p* value	aHR	*p* value
Age ≥ 56 anni	56 (52.8%)	6 (20.7%)	1.59	**0.018**	1.14	0.631
Menopause	63 (59.4%)	8 (27.6%)	2.06	**<0.001**	1.97	**0.012**
HR+	68 (64.2%)	23 (79.3%)	0.76	0.180	0.68	0.104
HER2 3+ (IHC)	83 (79.8%)	23 (79.3%)	1.08	0.754	3.34	0.239
HER2 positive (FISH)	20 (19.3%)	5 (17.2%)	1.00	0.995	3.03	0.289
Ki67 ≥ 20%	86 (83.5%)	21 (75%)	1.32	0.299	1.38	0.259
Time elapsed from diagnosis to 1st therapy	1052 ± 1281	917 ± 1094	1.00	0.423	1.00	0.319
Visceral Metastases	68 (65.4%)	16 (57.1%)	1.14	0.517	0.97	0.898
Brain metastases	14 (13.2%)	1 (3.7%)	1.66	0.079	1.68	0.153
Adjuvant Trastuzumab	53 (52.5%)	13 (46.4%)	1.06	0.775	1.01	0.972

Legend: HR: hormone receptor.

For the analysis of Complete (CR)/Partial responses (PR), with respect to Stable Disease (SD), multinomial logistic regression estimated a significant adjusted association only with menopause (OR = 0.25, *p* = 0.011). Age ≥56 years old and brain metastases were significantly associated to Progression of Disease (PD) responses, with respect to SD (OR = 3.29, *p* = 0.002 and OR = 2.95, *p* < 0.001, respectively), while menopause almost reached the statistical significance (OR = 0.41, *p* = 0.052), see Table 4.

**Table 4:** Multinomial logistic analysis

	CR_PR				PD			
	Univariate analysis		Multivariate analysis		Univariate analysis		Multivariate analysis	
	OR	*p* value	OR	*p* value	OR	*p* value	OR	*p* value
Age ≥56 years old	0.73	0.382	2.10	0.185	1	1	3.29	**0.002**
Menopause	0.44	**0.028**	0.25	**0.011**	0.92	0.898	0.41	0.052
HR+	0.47	0.058	0.48	0.094	0.32	0.101	0.24	0.089
TD1T	1.00	0.324	1.00	0.936	1.00	0.384	1.00	0.349
Visceral metastases	0.71	0.357	0.75	0.503	0.79	0.768	0.94	0.94
Brain metastases	1.65	0.415	0.90	0.868	2.95	0.238	2.95	**<0.001**
Adjuvant Trastuzumab	1.51	0.267	1.78	0.150	1.80	0.395	2.94	0.075

Legend: CR Complete Response, PR Partial Response, PD progressive disease, HR: hormone receptor, OR: Odds ratio; TD1T: time from diagnosis to first line treatment; OR and corresponding p values are obtained using Multinomial logistic regression.

As regards to Ki67 >20%, HER2 3+ and HER2 2+ at IHC and amplified at FISH evaluation, univariate multinomial regression models using bias reduction methods estimated a non-significant association for both CR/PR and PD responses (OR = 2.20, *p* = 0.114 and OR = 7.14, *p* = 0.196, respectively, for Ki67 >20%; OR = 2.2, *p* = 0.114 and OR = 7.14, *p* = 0.196, respectively, for HER2 3+; OR = 1.07, *p* = 0.884 and OR = 0.94, *p* = 0.937, respectively, for HER 2+ amplified).

### Adverse events

Toxicity recorded was generally limited to grade one and two (Table 5). Only in one case grade 3 neutropenia was registered in a patient reporting a complete long-lasting response. Grade 2 transaminitis was reported in 13% of the cases. Most recorded toxicity was grade 1 raised liver enzymes in 25%, following by grade 1 asthenia in 21% of the cases. Grade 2 hematological toxicity, including neutropenia, anemia and thrombocytopenia, was reported in about 3% of the cases. Other grade 2 toxicity (mucositis and diarrhea) was reported in about 2% of the cases. All these adverse events did not require dosage modifications and were managed according to product information schedule.

**Table 5:** Adverse events on 135 patients

Grade	Neutropenia	Anemia	Thrombo cytopenia	Mucositis	Diarrhea	Transaminases	Asthenia	Neuropathy	Alopecia
1	6 (4%)	8 (6%)	8 (6%)	2 (1,4%)	2 (1,4%)	34 (25%)	29 (21%)	3 (2%)	5 (3,7%)
2	1 (0,7%)	2 (1,4%)	1 (0,7%)	0 (0)	0 (0)	18 (13%)	0 (0)	0 (0)	8 (6%)
3	1 (0,7%)	0 (0)	0 (0)	0 (0)	0 (0)	0 (0)	0 (0)	0 (0)	0 (0)
4	0 (0)	0 (0)	0 (0)	0 (0)	0 (0)	0 (0)	0 (0)	0 (0)	0 (0)

## DISCUSSION

In this study, a multicenter experience concerning 135 metastatic breast cancer patients treated with T-DM1 following first line docetaxel and double blockade with pertuzumab and trastuzumab was reported. This is the largest case series reported until now. The reference study is Emilia which reported an ORR of 43.6%, median PFS of 9.6 months and median OS of 30.9 with T-DM1 [2]. In this study, patients were treated in first line patients with a regimen now considered suboptimal (trastuzumab plus a taxane). In our study and in another real-life trial performed by Conte et al. on 77 patients [21] with comparable age and hormone receptor expression, ORR were lower: 20% and 27%, respectively. In [21] more patients (45.4% versus 35%) had metastatic disease at diagnosis and many patients (78.5% versus 48%) were treated with adjuvant trastuzumab. In the study by Conte et al., mPFS was 6.3 months and median OS was not reached at the data cut-off. In our study, mPFS was 10.5 months, therefore, closer to Emilia. This result proves that T-DM1 maintains activity and efficacy also after pertuzumab and trastuzumab.

Metastatic breast cancer is still an uncurable disease, therefore, prolonged disease control at expense of mild toxicity represents one of the most desired endpoints. A relevant clinical benefit by T-DM1 was found despite a tumor response rate not exciting in patients pretreated with pertuzumab [18]. In our report, clinical benefit rate reached 50%. About 44% of the patients were on trastuzumab emtansine for more than 1 year. This result was higher than about 17% reported in [21]. Despite quality of life was not formally assessed in our study, the clinical benefit reported and the low toxicity registered confirm that T-DM1 is a manageable and active drug.

In our study, menopausal status was found to significantly influence the risk of progression at multivariate analysis. This factor was previously recognized to influence cancer progression [22]. We also found that brain metastases and adjuvant trastuzumab significantly influence the risk of progression.

The role of trastuzumab as well as pertuzumab pretreatment in HER2 positive patients is not clearly defined.

Some studies seem to suggest lower efficacy of T-DM1 following the triplet. A recent Canadian study [23] report on 104 patients treated with T-DM1 at any line. Event Free Survival was significantly longer in the pertuzumab-naïve group compared with pertuzumab exposed group (median time to treatment failure [TTF] = 18.7 vs 5.5 months; *p* < .001). Similarly, overall survival was better in pertuzumab naïve cohort as compares to pertuzumab pretreated.

In the study by Fabi et al [24], patients with prior trastuzumab/pertuzumab had significantly worse PFS compared with 73 patients with prior trastuzumab only (5 versus 11 months). In a multicenter, Italian cohort of 250 patients, PFS and OS were numerically less for patients with prior trastuzumab/pertuzumab in comparison to patients with prior trastuzumab only [20]. Another study on 42 patients was in line with previous findings [16].

It appears quite predictable that pretreated patients fare worse than untreated as other studies document [25].

Interpretation for these results include selection bias, the lack of data on additional factors, which may condition a different outcome like performance status, comorbidities, disease burden. As comes from the PRAEGNANT Real-World Breast Cancer Registry [26], Higher Eastern Cooperative Oncology Group (ECOG) scores, negative hormone receptor status, and visceral or brain metastases were associated with more frequent use of the sequence pertuzumab-trastuzumab followed by T-DM1. Probably a better definition of prognostic factors at baseline could define which classes of patients had a poor outcome.

On the other hand, the choice of administer the best therapy first is increasingly preferred in a wide range of tumor and was supported in breast cancer [17]. Furthermore, a metanalysis confirms the use of T-DM1 in metastatic breast cancer whatever the line [27].

Before the publication of real practice studies on T-DM1 following the double blockade, few information was available because no prospective study has been projected. Nevertheless, T-DM1 is recommended by different guidelines as the standard second line therapy in metastatic HER-2 positive breast cancer [13–15]. The only study prospectively evaluating the sequence is the PERNETTA study [28], a non-comparative randomized open label phase II trial of pertuzumab + trastuzumab with or without chemotherapy both followed by T-DM1 in case of progression, in patients with HER2-positive metastatic breast cancer. However, this study is primarily focused on a first-line therapy without chemotherapy. Recruiting studies are searching on novel therapeutic combination including T-DM1 in advanced/metastatic setting. There is no scientific interest in prospectively set up clinical studies aimed to formally evaluate the sequence of triplet followed by T-DM1. Therefore, the only available data on this matter comes from real practice studies, which give an insight into the effectiveness reported in an unselected population.

Despite the increased rate of survival of metastatic breast cancer patients overall, a rate of patients is lost at any line of therapy. In two different studies concerning patients’ series treated predominantly before 2010, 3% and 26% of patients reached the goal of third-line treatment [17]. This evidence underscores the need to give to our patients the best treatment as early as possible [29]. Summarizing, the available evidence is substantially in favor of the choice of T-DM1 in treatment of HER2 breast cancer at second and later lines.

## MATERIALS AND METHODS

### Patients

Seven centers were involved in the retrospective collection of data concerning metastatic HER2 breast cancer patients treated with second line TDM-1 following trastuzumab plus pertuzumab and docetaxel. Inclusion criteria were: HER2 positive breast cancer, first line treatment with pertuzumab plus trastuzumab and a taxane, disease progression to first line, measurable documented disease. Patients who did not respect the criteria of sequence of double blockade as first-line treatment and second-line T-DM1 were excluded from the study. Patients were routinely treated with T-DM1 at standard dosage of 3.6 mg per kilogram of body weight intravenously every 21 days. All patients gave informed consent to data treatment to study purposes. Adverse events were monitored continuously and graded according to the CTCAE, version 3.0.

All patients were evaluated during treatment according to clinical practice routine assessment, including clinical examination and laboratory exams at every treatment administration, US/X rays and Computed Tomography any 3-4 cycles of therapy in absence of clinical disease progression.

Data collected were age, premenopausal status, basal information on Ki67 (nuclear antigen expressed in cycling cells) (categorized as ≥ or < 20%), hormone receptor expression and HER2, this latter by immunohistochemistry (IHC) and Fluorescent *in situ* hybridization (FISH) in case of equivocal expression. Despite the lack on concordance of the optimal value of Ki67 cut-off, 20% was chosen based on some suggestions in favor of this value as prognostic [30–32]. Moreover, database included information on the use of trastuzumab as adjuvant therapy, sites of metastatic disease, best response to first- and second-line chemotherapy (Table 1). The primary aim of the study was to evaluate the efficacy and activity of T-DM-1 in second line treatment; however, data concerning first line were also collected. ORR represents the sum of complete and partial responses on the total number of patients expressed as percentage. The clinical benefit rate obtained considering the complete, partial and prolonged disease stabilization (more than 6 months) on the total number of patients was also evaluated.

### Immunohistochemistry and FISH

IHC was performed locally to assess Estrogen (ER) and progesterone receptor (PgR), Ki-67 level (nuclear antigen expressed in cycling cells), and HER2 status. HER2-positive status was determined by means of immunohistochemical analysis (with 3+ indicating positive status) (HercepTest; Dako A/S, Glostrup, Denmark). When a result of 2+ at IHC staining was obtained, FISH was performed (with an amplification ratio ≥2.0 indicating positive status). FISH testing was performed using the PathVysion HER2 DNA Probe (Abbott Molecular, Abbott Park, IL) according to previously defined protocols. As concerns Ki 67, IHC reaction was performed with monoclonal antibody against human Ki-67 (Clone 30-9, Ventana Medical Systems, Inc., Tucson, AZ, USA) and ultraView Universal DAB Detection Kit (Ventana Medical Systems, Inc., Tucson, AZ, USA). For Ki-67, nuclear expression was recorded quantitatively and categorized as ≥ or < 20%.

### Statistical analysis

Data were presented as median [min; max] for quantitative variables, and as number of patients (%) for qualitative variable.

Progression free survival calculated as the time from the start of treatment to disease progression or death was evaluated. Kaplan–Meier was used to assess the PFS during follow-up. Univariate and Multivariate Cox proportional hazard regression analysis was performed to estimate the Hazard Ratios for PFS, and a Forest plot was used to display the results.

Multinomial logistic regression was used to model responses and test for significant predictors. We chose SD as reference category; thus, results should be interpreted as the ratio of the odd of choosing CR-PR or PD over the odd of choosing SD. Since for Ki67 >20%, HER 3+ and HER 2+ amplified there was a strong imbalance of the events across categories, which may arise a separation problem in logistic regression, we applied univariate multinomial regression analysis using bias reduction methods [33] to model these three latter variables.

All statistical analyses were performed using R 3.6.0 software. Survival analysis was performed using survival package, version 2.44–1.1. Multinomial logistic regression was performed using nnet package, version 7.3–12. multinomial regression models using bias reduction method was performed using brglm2 package, version 0.6.1. Statistical significance was predetermined as *p* < 0.05.

## Abbreviations

HER2: Human Epidermal Growth Factor Receptor 2
FISH: Fluorescent in Situ Hybridization
T-DM1: adotrastuzumab emtansine
FDA: Food and Drug Administration
MDR: multidrug resistance
ORR: Overall Response Rate
CR: Complete Response
PR: Partial Response
SD: Stable Disease
DCR: disease control rate
CBR: clinical benefit rate
PFS: progression-free survival
OS: overall survival
IHC: immunohistochemistry
FISH: fluorescence *in situ* hybridization
RECIST: Response Evaluation Criteria in Solid Tumors
